# Mortality and Prevalence of Typhoid Fever

**Published:** 1913-05

**Authors:** Lewis M. Gaines

**Affiliations:** Atlanta, Ga.


### MORTALITY AND PREVALENCE OF TYPHOID FEVER.

By Lewis M. Gaines, B. S., M. D., Atlanta, Ga.

The subject for consideration demands a more or less statistical discussion from which certain conservative conclusions may be drawn. The proverbial dryness of such a report, however, I hope to enliven by the very practical nature of the inferences to be drawn.

The latest available statistics upon which this paper is based are three years old with the exception of the local figures here in our own city. The bulk of the statistics I obtained from the Ninth Annual Report on Mortality Statistics issued by the Department of Commerce and Labor at Washington, which includes full reports through 1908. These reports are compiled from that portion of the United States known as the Registration Area which unfortunately represents only a moderate portion of the entire country. Certain of the figures have been obtained from other reliable sources, which I will be glad to give upon request.

### General Prevalence.

Typhoid fever, which only since 1829 has been recognized as a definite clinical entity is widely distributed throughout the world, but prevails with greater relative frequency in temperate climates. Each added ray of light, which has been cast upon the etiology of the disease bears out the aphorism originated by Osler some years ago that the disease is every where an index of the sanitary intelligence of a community. I think, however, the fault is not so much with the intelligence of fever infested communities as it is with the lack of efficiency in carrying out measures which are perfectly intelligible, and known to be necessary and prophylactic. There is a certain lassez faire attitude adopted frequently which views with perfect complacency an excessive mortality from all preventable diseases. Such an attitude and such a condition of inefficiency spring from several sources. A study of the prevalence of the disease leads me to recognize some of the following: (1) Political machinations which in some cities have persistently thwarted efforts to revolutionize sanitary arrangements. (2) especially in military circles the tedious process known as red tape which was very efficient in promoting the typhoid mortality at Chicamauga and other encampments during the Spanish-American War. (3) failure to enlist a well organized public sentiment which will demand a thorough institution of the proper measures and a system of inspection which will keep such measures efficient. (4) lack of authority on the part of those constituted to prosecute the work of sanitation—sometimes, perhaps, lack of efficiency, and here, too, politics may have had a hand.

I wish, at this point, to quote a few sentences from an article on the House Fly, by N. A. Cobbs, recently appearing in the National Geographic Magazine, and which is very applicable. Says he: "In sanitary matters the tragedy that appeals to us strongly enough to make us do something worth while must be a tragedy quick in its action and very awful in its results. One is almost tempted to say that if only a disease is insidious enough it may proceed without opposition, even though we know all about its cause and the means for its prevention."

I now wish to present some concrete evidence which to our country, and to our city is in the nature of an indictment.

According to the census report of 1900, typhoid fever occupied fourth place in the mortality list, causing 35,379 deaths. In England and Wales, only 3,347 persons died of the disease during the same period. We had the distinction of having a death rate nearly three times that of our English cousins.

Turning now to the 1908 mortality statistics, limited to the registration area, we find that in the year 1908, 11,375 deaths occurred from typhoid fever, or 25.3 per 100,000 population, which figure was a great improvement over that of the preceding year which was 30.3 per 100,000. The average from 1901 to 1905 was 32.2 per 100,000.

Compare these figures with those of other countries. The average death rate from typhoid fever in the United States is nearly three times that of England and Wales, as already stated, where the rate is 11.2 per 100,000, double that of such a densely populated country as Belgium which has a rate of 16.8 per 100,000, and over four times that of the German Empire where the rate is only 7.6 per 100,000.

Turn now to the death rate in individual cities, and note the comparisons. From the year 1881 to the year 1885 the London death rate was 23 per 100,000. In 1908 the rate was only 5 per 100,000. Note the result not only of the knowledge of prevention, but of the application of that knowledge. In Edinburg from 1881 to 1885 the average annual death rate was 29 per 100,000. In 1908 it was 2 per 100,000. Thus with the growth not alone of a sanitary intelligence, but as the result of the actual daily enforcement of the proper measures have such remarkable results been achieved. Now turn to our cities. The most encouraging results have occurred in New York City, where the death rate from 1881 to 1885 was 40 per 100,000. In 1908 it had fallen to 12 per 100,000. In Philadelphia, for many years a fever smitten city, the average death rate from 1881 to 1885 was 69 per 100,000. In 1908 it was 35 per 100,000, a rate in excess of that for London and

Edinburg twenty years ago. And yet one hears how progressive a people are the Americans.

Let us come closer home and hear the figures for the Southern Cities and our own city.

| | 1904 | 1905 | 1906 | 1907 | 1908 |
|---|---|---|---|---|---|
| Birmingham | | | | | 107.6 |
| Montgomery | | | | | 58.3 |
| Jacksonville | 85.5 | 93.5 | 71.7 | 155.1 | 104. |
| Savannah | 78.8 | 40.1 | 40.8 | 77.3 | 22.5 |
| Atlanta | 60.8 | 70.1 | 75.2 | 102.5 | 68.5 |

To further emphasize the matter this statement appears in the Mortality Statistics: "An excessive death rate from typhoid fever appears year after year in a number of cities as follows:" I enumerate only the Southern cities, Jacksonville, Atlanta, Nashville, Lynchburg and Petersburg.

Mr. Thornton, Clerk to the city Board of Health has kindly made out the following tabulation for me:

| | Cases Reported | Deaaths Reported |
|---|---|---|
| 1905 | 117 | 66 |
| 1906 | 161 | 64 |
| 1907 | 314 | 86 |
| 1908 | 132 | 66 |
| 1909 | 149 | 65 |
| 1910 | 169 | 66 |
| 1911 | 315 | 90 |
| 1912 | 268 | 59 |

A study of these figures leads to several inevitable conclusions: (1) The prevalence and mortality from typhoid fever in Atlanta is not only excessive but continuously so. Exactly the same number of deaths were reported in 1905 as in 1910. (2) It is very evident that approximately a third only of the cases which occurred were reported. The figures given would indicate a mortality of about 30 to 50 per cent. The mortality could hardly have been 10 per cent, and probably less. This has bearing on the spread of the disease. (3) The growth of the city must be taken into account. We had a greater population in 1910 than in 1905, yet the same number of reported deaths.

This may mean a real proportionate decrease or inaccurate returns.

With the installation of the new sewage system and water purification plant we may look for a real decrease in the number of cases. The milk situation is I believe being handled better than ever, and the flies are receiving well merited attention. So that I think we may feel optimistic. But the efforts must be continuous and unceasing.

The experience of Cincinnati is interesting as regards the the bearing of a purification plant on the prevalence of typhoid fever. During the first five months of 1906 there were in that city 1447 cases reported with 182 deaths, during the first five months of 1907 902 cases and 127 deaths. Then came the installation of the purification plant. Following this measure during the first five months of 1908 there were only 136 cases and forty-four deaths reported.

The effect of unsanitary surroundings upon the prevalence of typhoid fever can nowhere be better demonstrated than by the experiences of armies. The disease occurs especially in standing camps rather than among troops on the march. During the Spanish-American war nearly one-fifth of the soldiers in the encampments had typhoid fever. In the South African war more men died of typhoid fever than of wounds received in battle. It remaiend for the Japanese army to give the world a demonstration of the efficiency of sanitary measures when properly and rigidly carried out. In seven months only 133 cases of typhoid fever occurred.

I would call attention to the prevalence of typhoid fever at summer resorts. As a rule, summer resorts, open as a rule only for a limited time, are very deficient of sanitation. Sewerage is defective, water supplies untested and dangerous and flies in abundance. The same is true of farm houses, taking summer boarders. How often is it true that a family goes off in the summer in search of health or renewed vigor and returns laden with this disease and sometimes others as well. This occurs in the observation of every practicing physician, and will occur many times this very summer.

There are several other points of interest as regards the prevalence of the disease. It occurs with about equal frequency in men and women. It is a disease of youth and early adult life, by far the greater number of cases occurring between the ages of twenty and thirty. It may however occur at any age.

Season has a marked effect upon the prevalence of the disease. Almost without exception the greatest number of cases are observed in the late summer and early autumn. Cases occur, however, throughout the year, and oft-times there is an increase noted at certain times in both winter and spring.

MORTALITY.

Typhoid fever is not an excessively fatal disease, otherwise few would be left to populate the earth, or rather the United States, and most especially some of our thriving Southern cities.

According to Osler, who has given the subject exhaustive study, the mortality in private practice varies from 5 to 12 per cent and in hospital practice from 7 to 20 per cent. In some epidemics the mortality is very low, in others very high. During the small epidemic which occurred several years ago at one of our Colleges for Women near Atlanta, there were forty cases and no deaths. These cases were treated by a large number of physicians in various parts of the country, and many of the patients took railway journeys of considerable length, a proceedure not conducive to the best results.

In large epidemics a mortality of 5 per cent. may be considered a minimum. At times the mortality may slightly exceed twenty per cent as in South Africa where it reached 20.9 per cent.

A difference in mortality is often noted in different geographical regions. Thus it has frequently been observed that the disease is more fatal in mountainous sections than in low countries. This has been a matter of personal observation.

There is also a marked variation in the mortality of the disease in different families. Some families always have the disease very lightly, others very severely with many deaths.

In the case of one family I have in mind, father, mother and six children were all wiped out by the disease.

The conclusions to be drawn from the facts and figures given are painfully obvious. We have to do with a serious, expensive, yet preventable disease. That it can be almost eliminated as a mortality factor has been demonstrated by such municipalities as Hamburg, London and Edinburg. That such necessary and adequate measures have not been taken in the United States has been very sucessfully demonstrated especially by such cities as Jacksonville, Birmingham, and Atlanta.

1314 Empire Bldg.

POSTSCRIPT.

This paper was read two years ago before the Fulton County Medical Society in connection with a symposium on Typhoid Fever. Since it was written considerable new statistical material has become available and the prophylactic vaccination against typhoid fever has been perfected. Thus far vaccination has had little effect in reducing the prevalence of the disease in civil practice, as relatively only a small number of persons voluntarily availed themselves of its distinct protection. I have inserted in the paper the 1911 and 1912 statistics of the city of Atlanta. As stated, the number of cases reported is misleading. Probably, judging by the known mortality, there were twice as many cases as reported. The fact still remains that Atlanta is far behind other enlightened communities in sanitary intelligence and efficiency if the occurrence of typhoid fever is to be regarded as an index. (May 31, 1913.)

---

### SURGICAL SUGGESTIONS.

When operating upon a carcinoma of the stomach be sure to suture the abdominal wall with silk or linen. In these cases healing is very slow and the wound may burst open if absorbable sutures are used.—AMERICAN JOURNAL OF SURGERY

